# Cephalomannine inhibits hypoxia-induced cellular function via the suppression of APEX1/HIF-1α interaction in lung cancer

**DOI:** 10.1038/s41419-021-03771-z

**Published:** 2021-05-14

**Authors:** Asmat Ullah, Sze Wei Leong, Jingjing Wang, Qing Wu, Mohsin Ahmad Ghauri, Ammar Sarwar, Qi Su, Yanmin Zhang

**Affiliations:** 1 School of Pharmacy, Health Science Center, Xi’an Jiaotong University, Xi’an, Shaanxi 710061 P.R. China; 2 Department of Microbiology, Faculty of Biotechnology and Biomolecular Sciences, Universiti Putra Malaysia, Seri Kembangan, Malaysia

**Keywords:** Pharmacology, Cancer microenvironment

## Abstract

Lung cancer (LC) is one of the leading causes of cancer-related death. As one of the key features of tumor microenvironment, hypoxia conditions are associated with poor prognosis in LC patients. Upregulation of hypoxic-induced factor-1α (HIF-1α) leads to the activation of various factors that contribute to the increased drug resistance, proliferation, and migration of tumor cells. Apurinic/apyrimidinic endonuclease-1 (APEX1) is a multi-functional protein that regulates several transcription factors, including HIF-1α, that contribute to tumor growth, oxidative stress responses, and DNA damage. In this study, we explored the mechanisms underlying cell responses to hypoxia and modulation of APEX1, which regulate HIF-1α and downstream pathways. We found that hypoxia-induced APEX1/HIF-1α pathways regulate several key cellular functions, including reactive oxygen species (ROS) production, carbonic anhydrase 9 (CA9)-mediated intracellular pH, migration, and angiogenesis. Cephalomannine (CPM), a natural compound, exerted inhibitory effects in hypoxic LC cells via the inhibition of APEX1/HIF-1α interaction in vitro and in vivo. CPM can significantly inhibit cell viability, ROS production, intracellular pH, and migration in hypoxic LC cells as well as angiogenesis of HUVECs under hypoxia through the inhibition of APEX1/HIF-1α interaction. Taken together, CPM could be considered as a promising compound for LC treatment.

## Introduction

Lung cancer (LC) is the most common and deadly cancer that has recorded 2.2 and 1.8 million new cases and deaths, respectively, in 2020 worldwide^1^. Like other cancers, LC occurs due to the epigenetic and molecular deformities^2^.

Hypoxia plays a major role in the microenvironment of solid tumors. Intra-tumoral hypoxia has been associated with malignant phenotypes and poor prognosis, as well as with chemo- and radiotherapy resistance^3^. In mammalian cells, the main mediators of biological response to hypoxia stress are hypoxic-induced factor (HIF)-1α and HIF-2α. They dimerize with a stable HIF-1β subunit to form heterodimeric transcriptional factors that regulate many target genes^4^. Hypoxia response elements are involved in tumor origination, angiogenesis, and metastasis^5,6^. Stability of HIF-1α modulates glycolysis and affects tumor progression, energy production, intracellular acidosis, and angiogenesis. Intracellular acidosis is necessary to be avoided for the conservation of cellular homeostasis. Carbonic anhydrase 9 (CA9) as one of the key target genes activated by HIF-1α can neutralize intracellular acidosis^7^. During hypoxia, the enhanced stability of HIF-1α increases the expression of C–X–C chemokine receptor type 4 (CXCR4) and erythropoietin EPO, which are the main regulators of metastasis and angiogenesis in cancer^8,9^. HIF-1α is a vital transcriptional mediator that plays an important role in the alteration of tumor cells in response to hypoxia by regulating multiple cytokines that enhance the proliferation and angiogenesis of LC.

Apurinic/apyrimidinic endonuclease-1 (APEX1) is an important redox regulator that could reduce the oxidized cysteine into specific transcriptional activators^10^. Besides, APEX1 also regulates HIF-1α and nuclear factor kappa B, as well as the signal transducer and activator of transcription-3, which involved in the regulation of several key cellular functions, including DNA repair and angiogenesis^11,12^. Since APEX1 is highly expressed in cancer cells and has been served as a biomarker for LC and other cancers, targeting APEX1 is therefore a promising strategy in the search of new anticancer treatments^13–15^. On this account, further understanding of the crosstalk between HIF-1α and APEX1 would bring new insights into the development of novel treatment.

Cephalomannine (CPM) (Fig. S1A) is a natural compound isolated from *Taxus wallichiana* (yew species), which exhibited antitumoral activity^16^. In this study, we demonstrated that CPM inhibits APEX1/HIF-1α interaction, as well as their targeted hypoxia-induced genes including CA9, CXCR4, and EPO, which leads to the inhibition of intracellular reactive oxygen species (ROS), pH, migration, and angiogenesis of hypoxic LC cells. Furthermore, the inhibitory effects on APEX1 and HIF-1α of CPM were confirmed in vivo.

## Results

### CPM suppressed the proliferation of LC cells in vitro

**Fig. 1:**
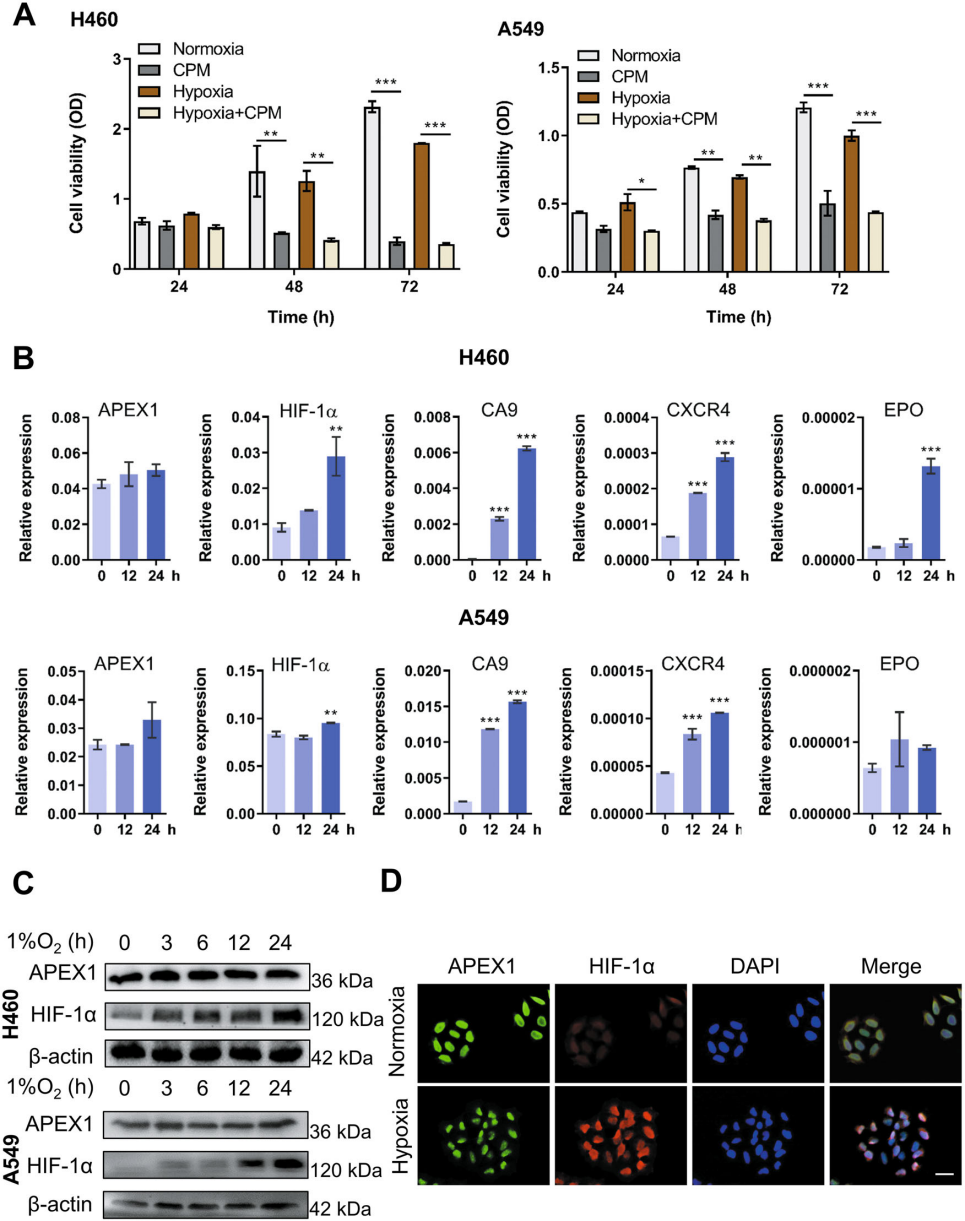
Cephalomannine (CPM) inhibits cell growth and hypoxia-induced APEX1/HIF-1α pathways in hypoxic LC cells. **A** H460 and A549 were treated with and without CPM under normoxia and hypoxia, and cell viability was examined at 24, 48, and 72 h. Data were expressed as mean ± SEM (*n* = 3). *P < 0.05, **P < 0.01, ***P < 0.001 compared with control cells. The results shown are representative of three independent experiments. **B** Hypoxia-induced APEX1/HIF-1α pathways in LC cells in vitro. Cell lines were cultured in normoxia and 1% O_2_ for 0, 12, and 24 h. HIF-1α, APEX1, CA9, CXCR4, and EPO mRNA expression values were assessed by RT-qPCR. Gene expression is normalized to ACTB. Data were expressed as mean ± SEM (*n* = 3). *P < 0.05, **P < 0.01, ***P < 0.001 compared with control cells. **C** H460 and H1299 cells were incubated in 1% O_2_ for 0, 12, and 24 h. Protein levels of HIF-1α and APEX1 were determined by western blotting. β-Actin were used as a control for western blots. *n* = 3. **D** H460 cells were cultured under hypoxia. APEX1 (green), HIF-1α (red), DAPI (blue) staining, and 3-channel merged images indicated the nuclear colocalization of HIF-1α. Scale bars, 50 μm. Data presented are representative of three independent experiments.

Firstly, the cell proliferation of several LC cell lines treated with CPM was investigated. Under normoxia, CPM significantly inhibited the proliferation of H460, A549, and H1299 cells with the IC_50_ values of 0.18, 0.20, and 0.37 µM, respectively (Fig. S1B). Since hypoxia is one of the key features of LC microenvironment contributing to the stabilization of HIF-1α and is responsible for the tumorigenesis of the hypoxic LC cells^17^, we therefore treated H460 and A549 cells with CPM for 24, 48, and 72 h, and the cells were cultured in a hypoxia chamber that offers stable 1% O_2_ environment, and the cell viability was determined. As shown in Fig. 1A, CPM could also significantly inhibit cell viability under hypoxia.

### CPM inhibited hypoxia-induced APEX1/HIF-1α expression and interaction

To confirm the clinical relevance of APEX1/HIF-1α pathway in LC, mRNA data from The Cancer Genome Atlas (TCGA) database in both lung adenocarcinoma and squamous cell lung carcinoma were analyzed. The results obtained and published studies^18^ showed that APEX1, HIF-1α, and the targeted genes (CA9, CXCR4, and EPO) are highly expressed in tumors in comparison to that of adjacent normal tissues (Fig. S2). Since hypoxic environment has been reported to promote tumorigenesis by elevating hypoxia-induced genes and proteins^19^, we therefore examined the expression of HIF-1α and APEX1 at both transcriptional and translational levels under hypoxic condition. We exposed H460 and A549 cells to 1% O_2_ for various time periods, and the relative gene expression of HIF-1α was significantly elevated, while only a slight increase of APEX1 gene expression was observed. Similarly, protein expression analysis by western blotting has confirmed the enhanced levels of HIF-1α in hypoxic LC cells (Fig. 1B, C). As shown in Fig. 1B, the expression of HIF-1α target genes, including CA9, CXCR4, and EPO, was also increased under hypoxia. As a transcriptional factor, HIF-1α transcriptionally activates various catalog of genes, including migration and metastasis factors after translocation to the nucleus. Immunofluorescence results showed the colocalization of HIF-1α and APEX1 in the nucleus under hypoxia (Fig. 1D).

**Fig. 2:**
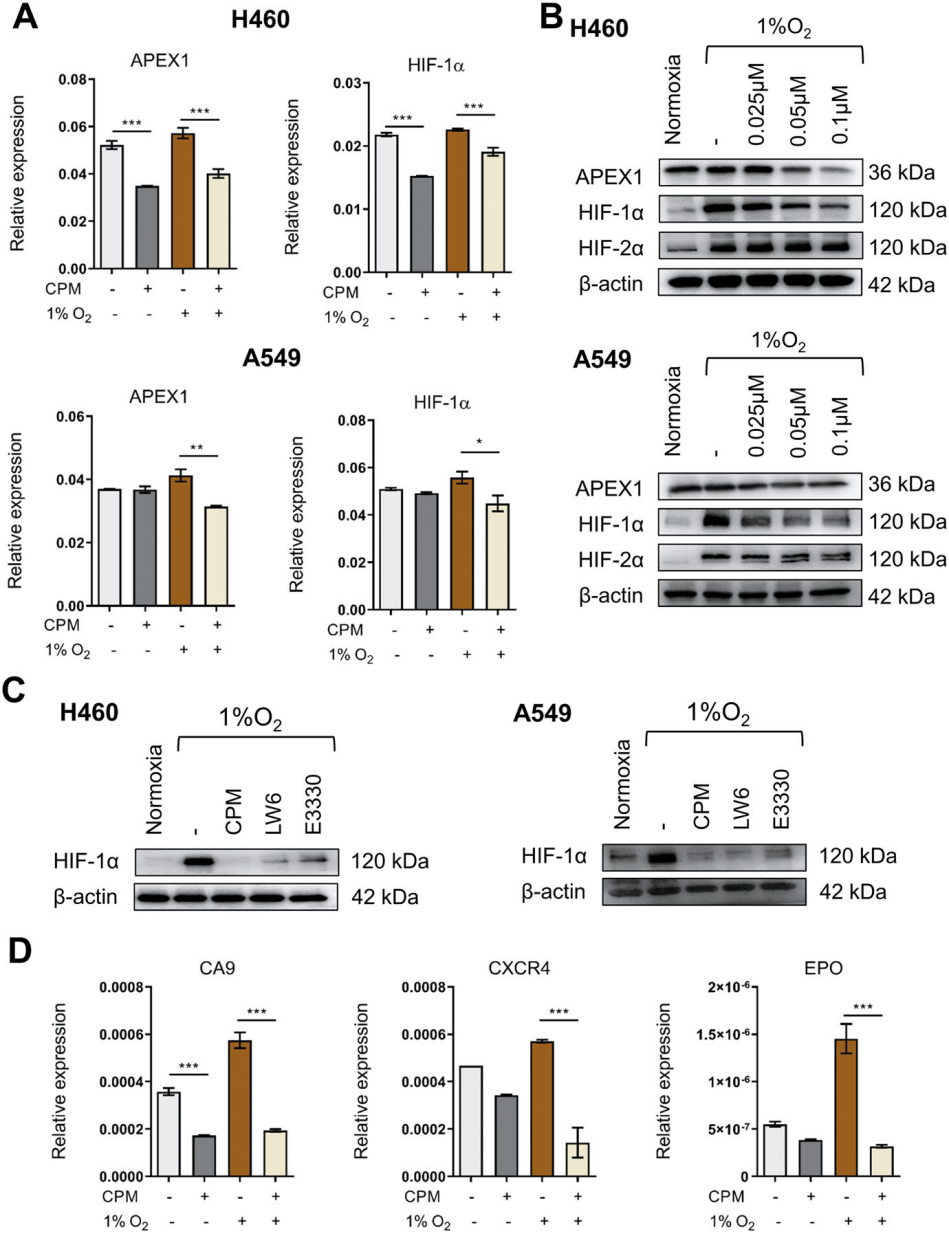
CPM inhibited APEX1/HIF-1α expression in hypoxic LC cells in vitro. **A** RT-qPCR analysis of APEX1 and HIF-1α expression in H460 and A549 cells with or without CPM during hypoxia and normoxia. **B** H460 and A549 cells were treated with CPM and cultured under 1% O_2_. HIF-1α and APEX1 protein levels were determined by western blotting. β-Actin was used as a control. **C** H460 cells were treated with CPM, 10 μM APEX1 inhibitor E3330, and 5 μM LW6 in normoxia and hypoxia. Protein expression of HIF-1α was assessed by western blotting. **D** Relative expression of CA9, CXCR4, and EPO in H460 cells treated with CPM in normoxia and hypoxia. Data were expressed as mean ± SEM (*n* = 3). *P < 0.05, **P < 0.01, ***P < 0.001 compared as indicated.

Next, the effects of CPM on the APEX1/HIF-1α interaction were evaluated in CPM-treated hypoxic LC cells. As shown in Fig. 2A, the relative expressions of APEX1 and HIF-1α were significantly inhibited after 24 h of treatment with CPM in H460 and A549 cells. Their corresponding immunoblotting analyses showed that treatment with CPM decreased the expressions of APEX1 and HIF-1α but not HIF-2α (Fig. 2B). To further identify whether APEX1 regulates HIF-1α at protein level under hypoxia, an inhibitor of HIF-1α, LW6, and an inhibitor of APEX1, E3330, were used to treat LC cells. Based on the results obtained, CPM, LW6, and E3330 have significantly inhibited the expression of HIF-1α at the protein level in hypoxic LC cells (Fig. 2C). The suppressed gene levels of CA9, CXCR4, and EPO also confirmed the suppression of HIF-1α signaling in CPM-treated LC cells (Fig. 2D).

**Fig. 3:**
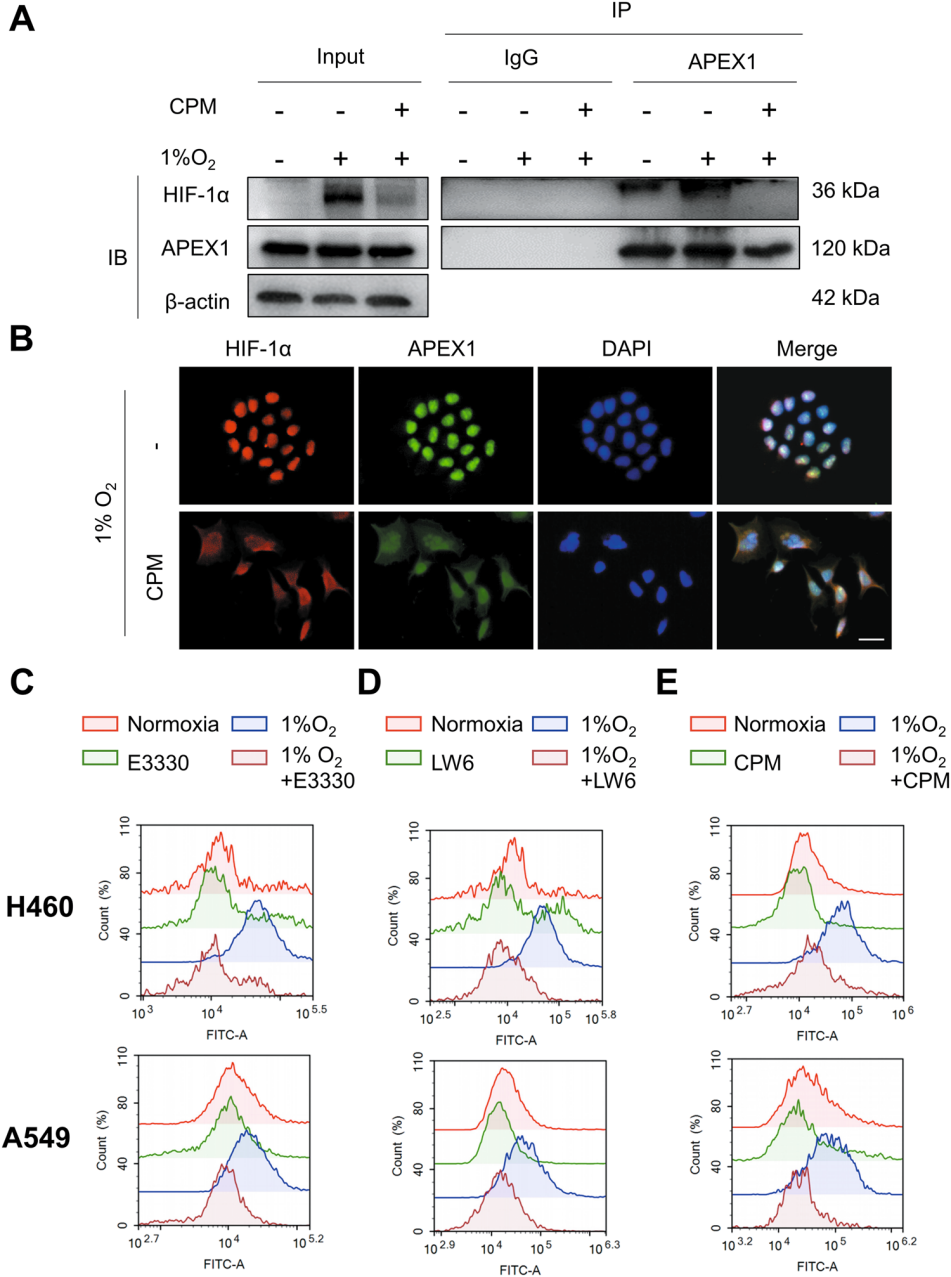
CPM inhibited APEX1/HIF-1α interaction and reactive oxygen species (ROS) in LC cells. **A** Cell extracts from H460 cells treated with CPM under hypoxia were immunoprecipitated with anti-APEX1 antibody or IgG. The IP complex was analyzed for HIF-1α or APEX1 by western blotting. **B** H460 cells were treated with CPM under hypoxia. Intracellular localization of APEX1 and HIF-1α was examined by immunofluorescence. APEX1 (green), HIF-1α (red), DAPI (blue) staining, and 3-channel merged images indicated the nuclear colocalization. **C** H460 and A549 cells were treated with E3330 under normoxia and hypoxia. **D** H460 and A549 LC cells were treated with LW6 under normoxia and hypoxia. **E** LC cells H460 and A549 treated with CPM under normoxia and hypoxia. Cells were stained with 10 μM DCFH-DA and measured with a flow cytometer.

Furthermore, hypoxic H460 cells were treated with CPM and co-immunoprecipitation results demonstrated that the interaction between HIF-1α and APEX1 occurred under hypoxia and was inhibited by CPM treatment (Fig. 3A). Moreover, immunofluorescence results showed the nuclear accumulation of APEX1 and HIF-1α which was disrupted by CPM treatment (Fig. 3B).

### CPM suppressed the intracellular levels of reactive oxygen species in hypoxic LC cells

Reactive oxygen species (ROS) is one of the major cellular mediators that is beneficial to an aerobic organism at the basal level^20^. However, it turns to be carcinogenic if excessive levels of ROS are persistently present in the organism^21^. Since APEX1 and stabilized HIF-1α have been reported to induce the upregulation of ROS^22^, we therefore evaluated the effects of LW6, E3330, and CPM on ROS released in hypoxic LC cells using the DCFH-DA probe. The results showed an increased level of ROS during the exposure of A549 and H460 cells to hypoxia. The levels of ROS were decreased with the treatment of E3330 and LW6, indicating that inhibition of APEX1 and HIF-1α within the cells led to the decreased levels of intracellular ROS (Fig. 3C, D). A549 and H460 cells treated with CPM also showed attenuated ROS levels within the cells (Fig. 3E). These results suggested that CPM might inhibit ROS levels via the control of APEX1/HIF-1α in hypoxic LC cells.

### CPM decreased intracellular pH in LC cells under hypoxia

**Fig. 4:**
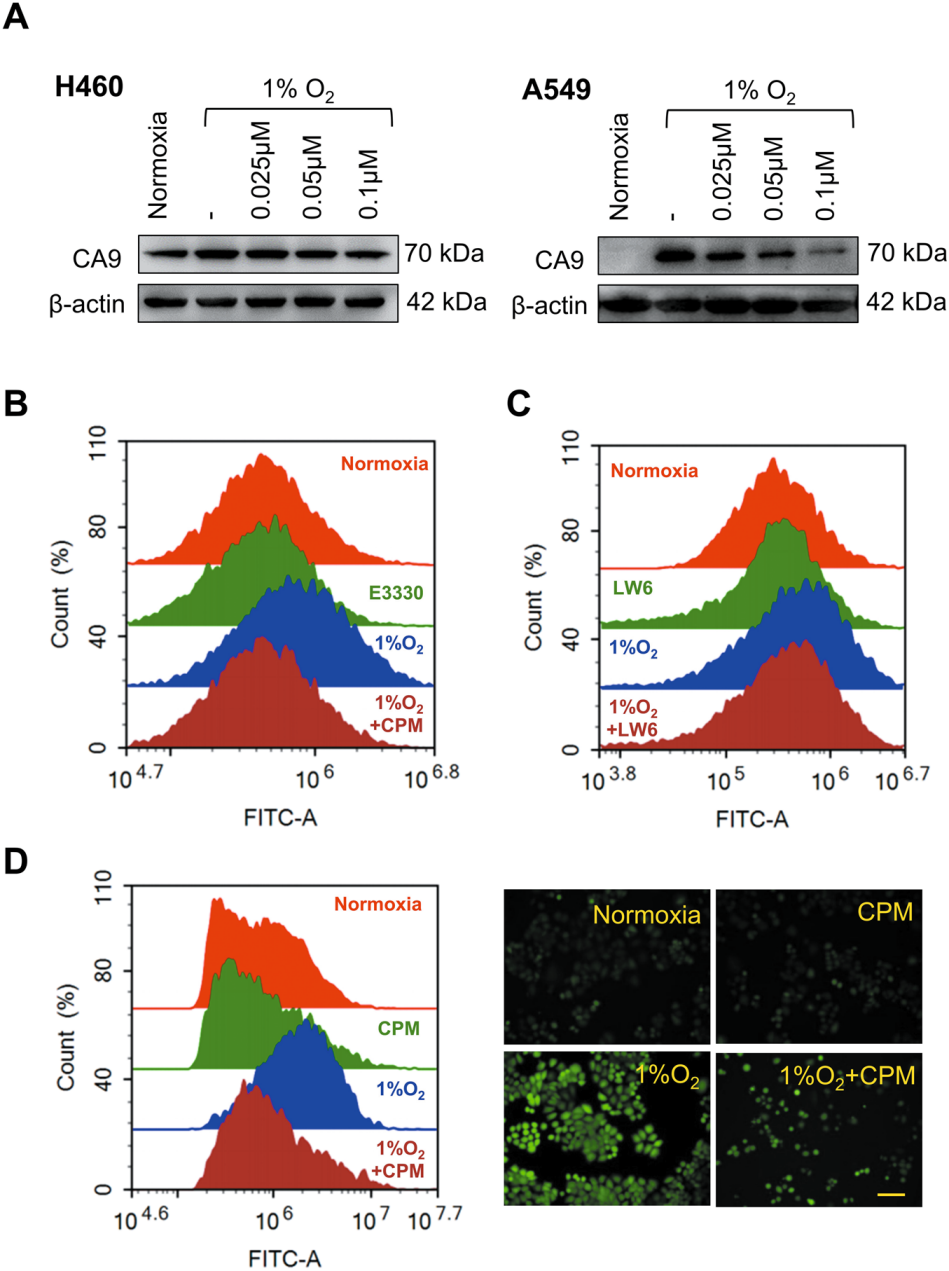
CPM could acidify intracellular pH in LC cells during hypoxia. **A** Western blot analysis of CA9 expression at various concentrations treated with CPM. **B** Intracellular pH determination of H460 cell line treated with APEX1 inhibitor E3330 under normoxia and hypoxia using flow cytometry. **C** Intracellular pH determination of H460 cells line treated with LW6 under normoxia and hypoxia using flow cytometry. **D** Intracellular pH determination of H460 cell line-treated CPM under normoxia and hypoxia using flow cytometry, using BCECF-AM dye for staining in H460 cells, images taken by a fluorescence microscope.

Acidification of extracellular pH and alkalization of intracellular pH are the hallmarks of tumor proliferation, invasion, metastasis, and aggressiveness^23,24^. CA9 plays an active role during intracellular pH regulation^25^. During hypoxia, CA9 catalyzes the conversion of CO_2_ to HCO_3_^−^ which functions to maintain the intracellular pH^26^. The treatment with CPM could inhibit the relative mRNA expression of CA9 which might affect the intracellular pH. Western blotting results confirmed that CPM can inhibit CA9 expression at various concentrations (Fig. 4A). Next, we measured the effects of CPM on pH regulation in H460 cells. The 24 h incubation in 1% oxygen is sufficient to stabilize the HIF-1α to induce the expression of CA9. After 24 h of culture, the cells were treated with E3330, LW6, and CPM for 24 h in a hypoxia incubator. E3330 and LW6 reduced the intracellular pH compared to hypoxic cells (Fig. 4B, C), indicating that inhibiting APEX1 and HIF-1α could reduce hypoxia-induced pH. CPM could greatly decrease the intracellular pH levels within hypoxia in H460 cells (Fig. 4D). Fluorescence images of H460 cells confirmed that CPM could acidify and bring stability in intracellular pH (Fig. 4D).

### CPM inhibited hypoxic-induced expressions of CXCR4, EPO, and MMPs

**Fig. 5:**
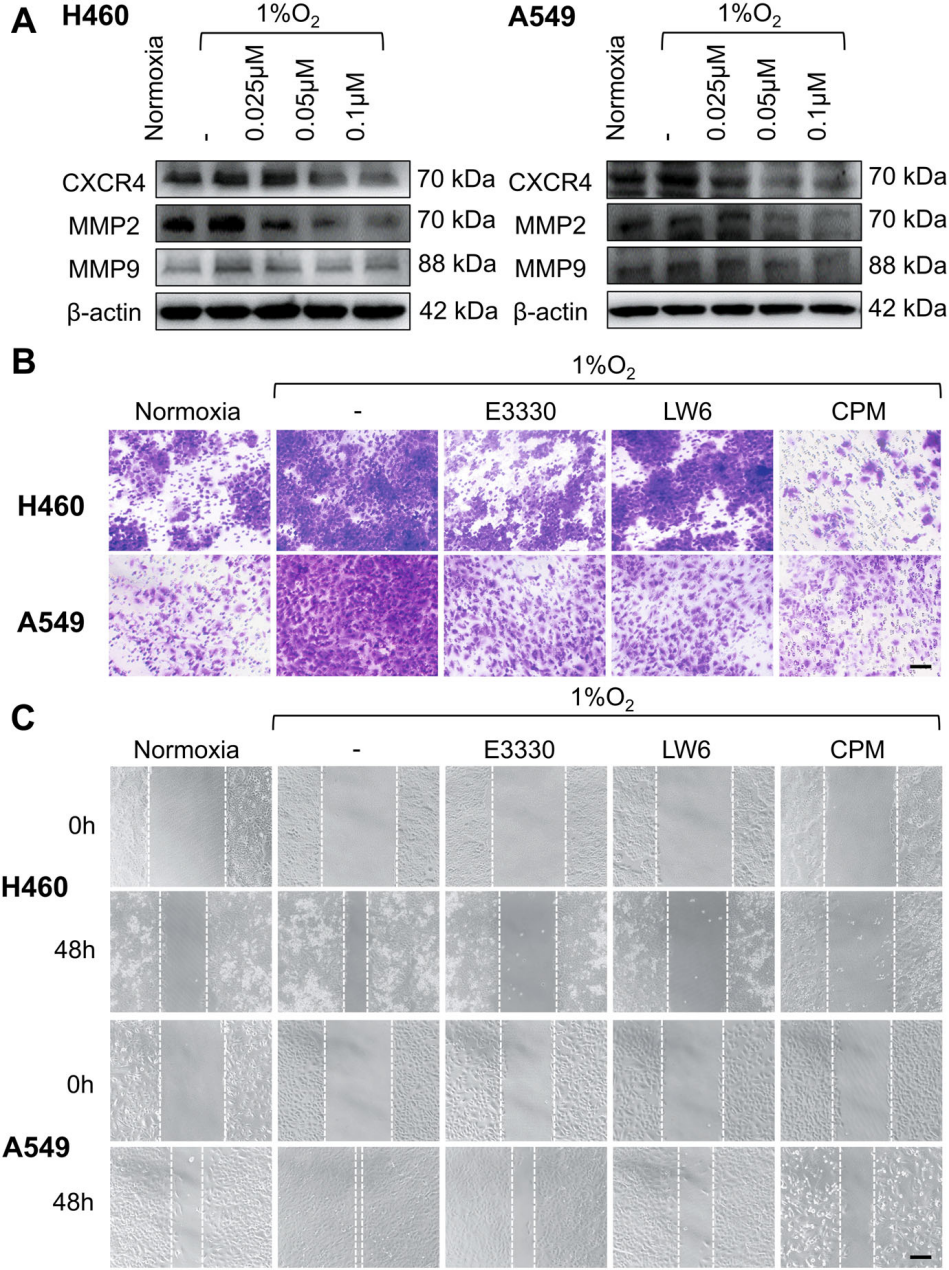
Inhibitory effect of CPM on migration in LC cells. **A** CXCR4, MMP2, and MMP9 expression was analyzed in hypoxic-exposed H460 and A549 cells and treated with CPM at various concentrations. **B** Hypoxia-induced migration in H460 and A549 cells was examined by transwell assay. H460 and A549 cells were cultured in the upper partition of a transwell chamber exposed to 1% O_2_, and treated with APEX1 inhibitor E3330, HIF-1α inhibitor LW6 and CPM. Scale bar, 50 μm. **C** Wound healing assay was performed to test the effect of CPM on cell migration. H460 and A549 cells were incubated with E3330, LW6, and CPM, and exposed to 1% O_2_ for 48 h. Scale bar, 100 μm. The results shown were representative of three independent experiments.

Hypoxic-induced CXCR4 and MMPs promote invasion, migration, and metastasis of tumors^27^. We determined the protein levels of CXCR4 and MMPs in hypoxic LC cells. As shown in Fig. 5A, the results of western blot analysis showed the inhibition of CXCR4, MMP2, and MMP9 proteins in CPM-treated H460 and A549 cells. To examine whether CPM can inhibit cell migration under hypoxia, we performed transwell assay. Indicated by migrated cell numbers, hypoxia increased migration of cells compared to normoxia, while E3330, LW6, and CPM could inhibit the migration during hypoxia in A549 and H460 cells (Fig. 5B). We further examined the effect of CPM on cell migration using wound healing assay, and the results confirmed that E3330, LW6, and CPM could inhibit cell migration under hypoxia (Fig. 5C). These data demonstrated that hypoxia increased cell migration and related protein levels, and CPM could inhibit the cell migration during hypoxia by acting on APEX1/HIF-1α pathway in LC cells.

### CPM potentially inhibited hypoxic-induced angiogenesis in human umbilical vein endothelial cells

**Fig. 6:**
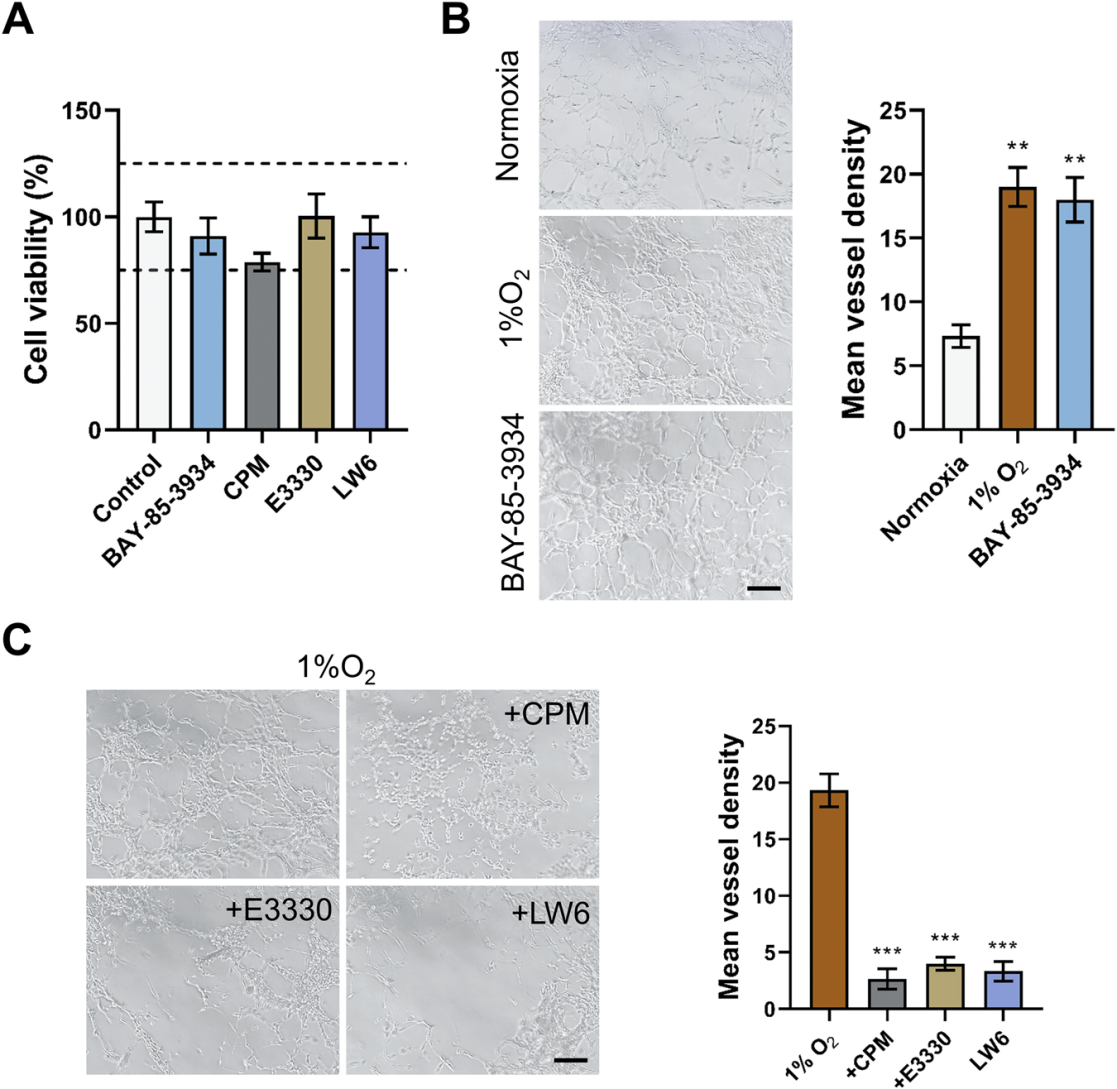
CPM inhibited the tube formation in HUVECs. **A** HUVEC cell viability assay by treating with 5 μM BAY-85-3934, 25 nM CPM, 10 μM E3330, and 5 μM LW6. **B** HUVECs cells were cultured in normoxia and exposed to 1% O_2_ and also treated with BAY-85-3934 in 1% O_2_. **C** One percent O_2_-exposed HUVECs were treated with CPM, E3330, and LW6. Scale bar, 100 μm. Data were expressed as mean ± SEM (*n* = 3). **P < 0.01, ***P < 0.001 compared to control groups.

Endothelial proliferation is one of the major factors of neovascularization-dependent condition during hypoxia^28^. To confirm this, we used a novel BAY-85-3935 agent to stabilize HIF-1α-induced EPO. Cell viability assay indicated that these compounds could not affect the cell viability of human umbilical vein endothelial cells (HUVECs) (Fig. 6A). BAY-85-3935 and 1% O_2_ significantly increased the vessel formation in HUVEC cells compared to normoxia (Fig. 6B). We then treated HUVECs with CPM, E3330, and LW6 within hypoxia. CPM, E3330, and LW6 significantly inhibited tube formation in HUVECs (Fig. 6C). Therefore, it was confirmed that CPM could inhibit angiogenesis of HUVECs during hypoxia.

### CPM inhibited the growth and APEX1/HIF-1α pathways in LC cells in vivo

**Fig. 7:**
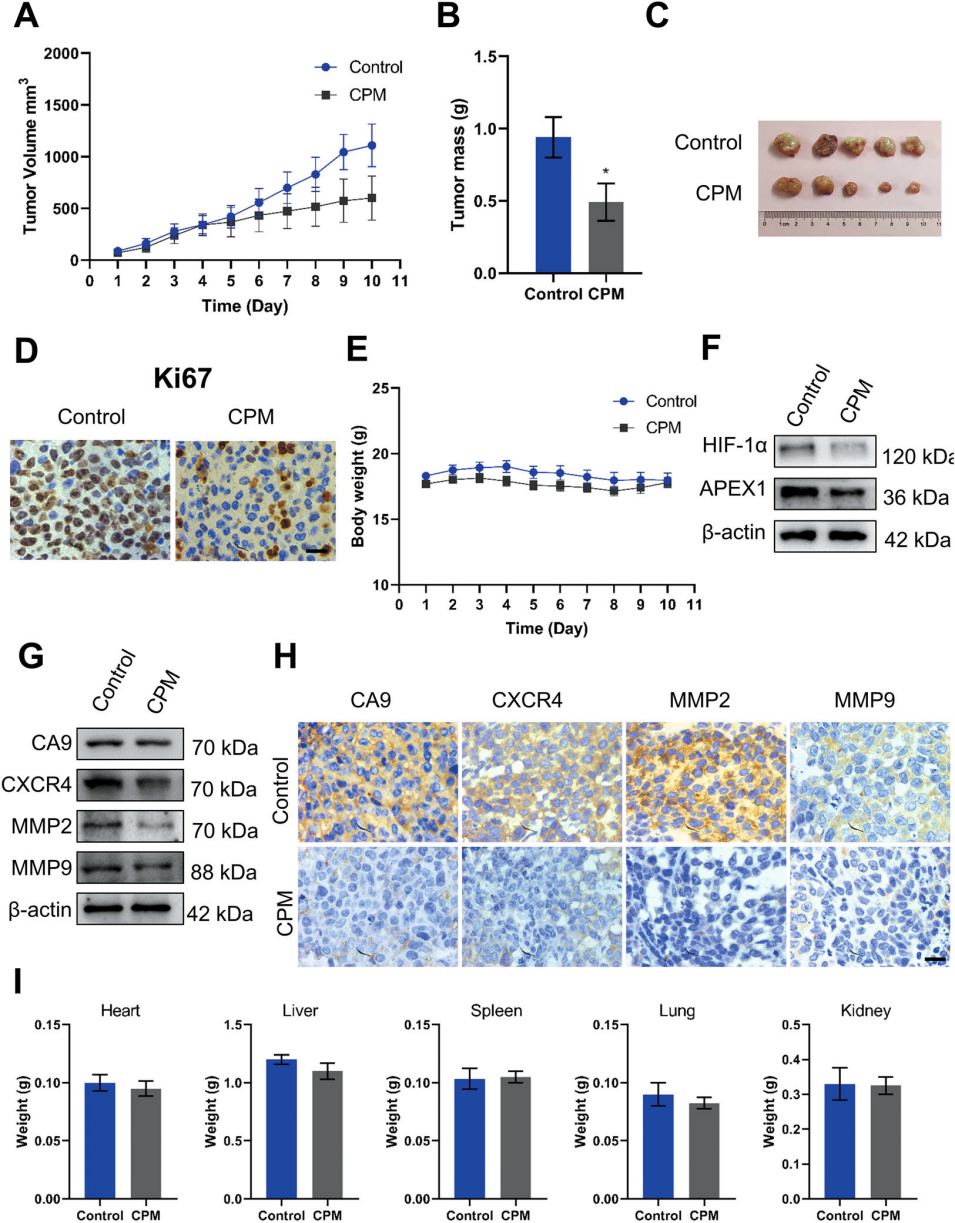
CPM inhibited growth and proliferation of H460 LC xenograft tumor model. **A** Xenograft tumor volume was measured in the control group and the CPM-treated group during the 10-day experiment. **B** Xenografts tumor mass was measured of control and CPM-treated group. **C** Image of tumors from the control group and the CPM-treated group in a xenograft tumor model. **D** Analyzing the tumor tissue proliferation, both control and CPM-treated tissue were stained with proliferation marker Ki67. Scale bar, 50 μm. **E** Xenograft animals body weight was measured in the control group and the CPM-treated group during the 10-day experiment. **F** Western blot analysis of APEX1 and HIF-1α in control group tumor tissue and CPM-treated tumor of xenograft tumor. **G** Western blotting analysis of protein expression of CA9, CXCR4, MMP2, and MMP9 in control group tumor tissue and CPM-treated tumor of xenograft tumor. **H** Immunohistochemical staining of CA9, CXCR4, MMP2, and MMP9 in the section of the control group and the CPM-treated group of xenografts nude mice. **I** Weight of the vital organs heart, liver, spleen, lung, and kidney of animals of the control group and the CPM-treated group. Data were expressed as mean ± SEM (*n* = 5). *P < 0.05, compared to control groups.

The effects of CPM on LC cells in vivo were observed using a nude mice xenograft model. H460 cells were subcutaneously implanted into nude mice. Then, mice were treated with either vehicle or CPM (0.4 mg/kg, i.p.) for continuous 10 days. Compared with vehicle treatment, CPM effectively reduced the volume and weight of H460 xenograft tumors (Fig. 7A–C). Consistent with our in vitro results, immunohistochemical staining data demonstrated that the expression of Ki67, a proliferation biomarker, was downregulated in response to CPM (Fig. 7D). No significant loss was detected in the body and organ weights of the experimental animals (Fig. 7E, I), suggesting that CPM significantly suppressed the growth of LC cell xenografts but had no major side effects in mice.

**Fig. 8:**
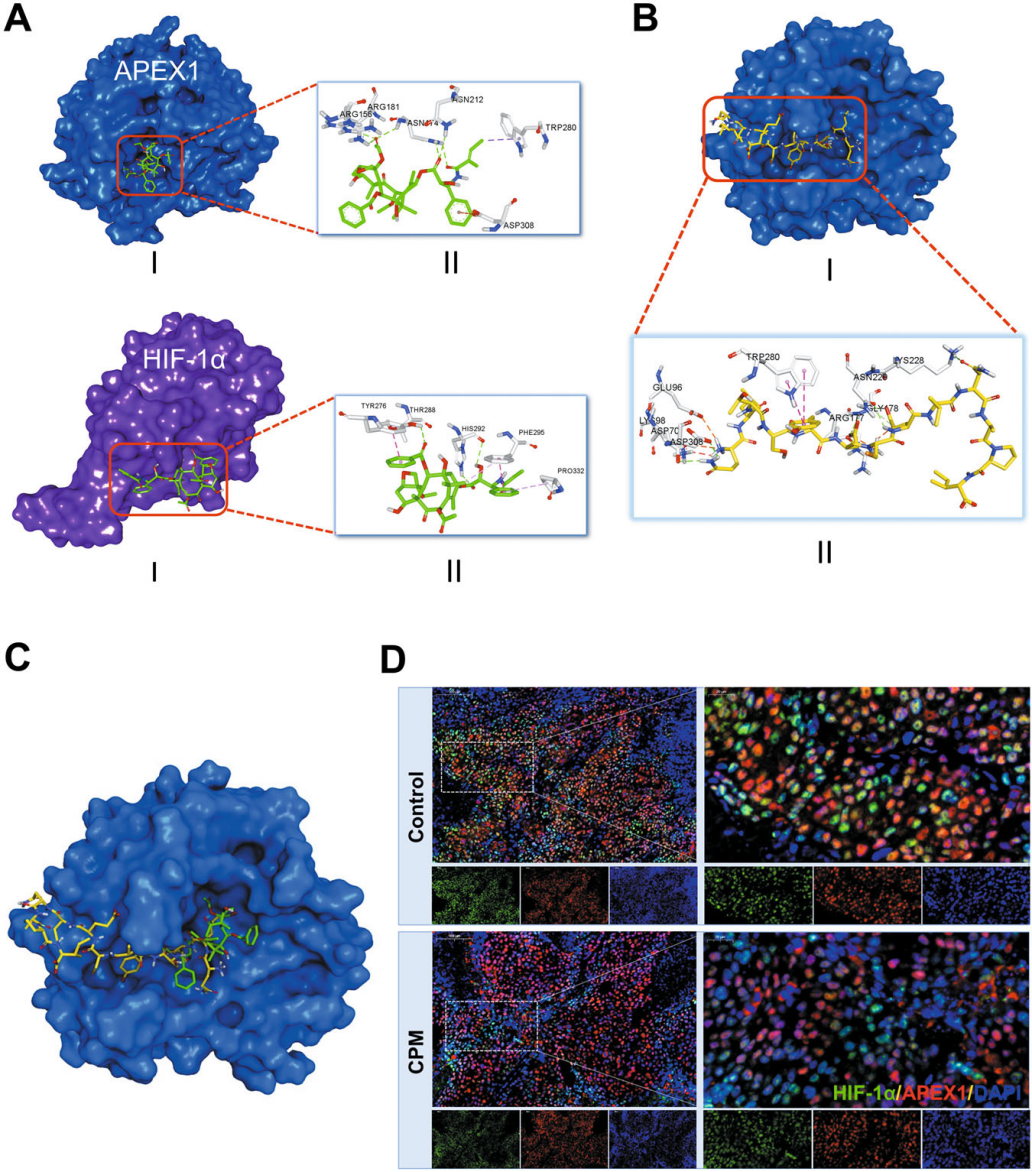
CPM inhibited hypoxic-induced HIF-1α/APEX1 interaction in H460 LC xenograft tumor model in vivo. **A** Molecular docking model of CPM with APEX1 protein (PDB: 3U8U) and HIF-1α protein (PDB: 4H6J). (I) 3D conformation position of CPM in the surface pocket. (II) Key interactions of CPM in the surface pocket. **B** Molecular docking model of HIF-1α peptide with APEX1 protein (PDB: 3U8U). (I) 3D conformation position of HIF-1α peptide in the surface pocket of APEX1. (II) Key interactions of HIF-1α peptide in the surface pocket of APEX1. **C** Overlapping of docking conformations of CPM and HIF-1α peptide in APEX1 protein. **D** Immunofluorescence staining of H460 tumor sections. Tumor section were stained with HIF-1α (green), APEX1 (red), and DAPI (blue).

Western blotting results confirmed that the expression of APEX1 and HIF-1α was inhibited in tumor tissue (Fig. 7F). Moreover, CA9, CXCR4, MMP2, and MMP9 protein levels were also reduced in CPM-treated tumors compared with control (Fig. 7G, H). This result is further supported by our molecular docking analysis (Fig. 8A) in which we found that CPM could interact with both APEX1 (−8.0 kJ/mol) and HIF-1α (−6.9 kJ/mol) surface pocket. In order to investigate the potential interactions between APEX1 and HIF-1α, as well as to explore the possibility of CPM in interrupting such interaction, molecular docking analysis was performed. Overlapping of docking conformations of CPM and HIF-1α peptide in APEX1 protein indicated that CPM might interrupt APEX1 and HIF-1α interaction via competitive binding with APEX1 (Fig. 8B, C). Immunofluorescence results of the tumor samples showed that CPM could inhibit the APEX1/HIF-1α colocalization and interaction in vivo (Fig. 8D).

## Materials and methods

### Cell lines and reagents

CPM (HPLC ≥ 98%, Lot: HC081430) was purchased from Baoji Kerui Biochemical Pharmaceutical Co., Ltd (Shaanxi, China). Human LC NCI-H1299, A549, and NCI-H460 cell lines were obtained from Shanghai Institute of Cell Biology in the Chinese Academy of Sciences. Passage numbers 20–40 were used. H1299 and H460 were cultured in RPMI-1640 medium with 10% fetal bovine serum (FBS) and A549 in Ham’s F-12 with 10% FBS, supplemented with 100 unit/mL penicillin and 100 mg/mL streptomycin at 37 °C in a humidified atmosphere of 5% CO_2_. Additionally, sodium pyruvate was added to Ham’s F-12. The 3-(4, 5-dimethylthiazol-2-yl)-2.5diphenyl-2H-tetrazolium bromide (MTT), trypsin, and dimethyl sulfoxide (DMSO) were purchased from Sigma-Aldrich (St. Louis, MO, USA). Penicillin and streptomycin were purchased from Harbin General Pharmaceutical Factory (Harbin, China) and North China Pharmaceutical (Shijiazhuang, China), respectively. FBS was purchased from Excell Bio (Shanghai, China). Crystal violet was purchased from Beijing Chemical Plant (Beijing, China). HIF-1α (20960-1-AP), APEX1 (10203-1-AP), HIF-2α (26422-1-AP), HIF-1β (14105-1-AP), CA9 (11071-1-AP), CXCR4 (11073-2-AP), MMP2 (10373-2-AP), MMP9 (10375-2-AP) rabbit antibodies, and β-actin (66009-1-Ig) mouse antibody were obtained from Protein Technology Group (Chicago, Illinois, USA). All the antibodies were used in the dilution of 1:1000 for western blotting. RIPA Lysis Buffer was obtained from Applygen Technologies (Beijing, China). Protease inhibitor cocktail and phosphatase inhibitor cocktail were purchased from Roche Technology (Basle, Switzerland). E3330 and LW6 inhibitors are purchased from Medchemexpress (NJ 08852, USA).

### Cell proliferation assay

The cells (1 × 10^4^ cell/well) were seeded in 96-well plates and incubated for 24 h. Different concentrations of CPM were added to the cells. The cells were again incubated for 48 h and 0.5 mg/mL MTT solution was added for 4–6 h at 37 °C incubation. One hundred and fifty microliters of DMSO were added and kept in a shaker for 15 min and plates were analyzed using a microplate reader (Bio-Rad, Hercules, CA, USA) at 490 nm of wavelength. Each experiment is performed in triplicate to determine the final value.

### TCGA database

We used public TCGA (http://cancergenome.nih.gov/) database to examine the expression of related genes in LC patients. We obtained the mRNA profiles of 533 adenocarcinoma and 59 non-tumor, as well as 502 squamous cell carcinoma and 49 non-tumor data, respectively. Significant difference was detected by *t*-test using graphpad prism (Prism 6.0, Graph Pad, La Jolla, CA, USA).

### Western blotting

After treatments, the cells were lysed in RIPA buffer containing protease and phosphatase inhibitors. The total protein was reckoned with BCA assay. Protein samples were subjected to SDS PAGE and transferred to PVDF membrane (Millipore, Bedford, MA, USA), which was then blocked with 5% non-fatty milk in TBST buffer and was incubated with the respective primary antibody at 4 °C for 6–8 h. Then the membrane was incubated with species-specific horseradish peroxidase (HRP)-conjugated secondary antibodies, and antigen–antibody complexes were imaged using the enhanced ECL kit. The images were taken using the Tanon5200 imaging system (Tanon, Shanghai, China). β-Actin was used as a control for normalizing protein loading.

### RNA isolation and RT-qPCR

RNA was isolated from CPM treated H460 and A549 cells in normoxia as well as hypoxia, according to the manufacturer’s protocol using an RNA extraction kit. Using Revert Aid First-Strand cDNA Synthesis Kit (Thermo Fisher Scientific, Waltham, MA, USA), the RNA samples were reverse transcribed. Quantitative PCR was performed using the iCycleri-Q real-time PCR detection system (Bio-Rad, Hercules, CA, USA). The primers with the following sequences were used: APEX1: 5′-CTGCTCTTGGAATGTGGATGGG-3′ and 5′-TCCAGGCAGCTCCTGAAGTTCA-3′, HIF-1α: 5′-CTCATCAGTTGCCACT TCCACATA-3′, and 5′-AGCAATTCATCTGTGCTTTC ATGTC-3′, CA9: 5′-ACCAGACAGTGATGCTGAGTG CTAA-3′ and 5′-TCAGCTGT AGCCGAGAGTCACC3′; for the quantification level, the ACTB method was used. The mRNA expression of each gene was normalized to ACTB; iCycler software was used for the calibration curve.

### Immunofluorescence

Cells were cultured as control and also treated with CPM in normoxia as well as hypoxia for 24 h, and the cells were fixed with 4% paraformaldehyde for 15 min. Cells were then kept in 0.1% Triton X-100 in PBS for 10 min. Then, they were washed three times with PBS and added 200 μL per well 0.1% BSA solution for blocking and incubated at 37 °C for 1 h. Then, the primary antibody of HIF-1α (mouse) and APEX1 (rabbit) was added and incubated for 4 h at 37 °C. It was then washed and added FITC-conjugated anti-mouse or anti-rabbit secondary antibodies for 1 h in the dark, and finally added DAPI for 10 min. The images were then taken using a fluorescence microscope.

### Measurement of intracellular ROS

The intracellular ROS were measured by the ROS assay kit. The cells were seeded in a six-well plate (1.5 × 10^5^/mL). After treatment, DCFH-DA (1 mL/well) was added with a final concentration of 10 μM in the dark, and the cells were incubated for 20 min at 37 °C. Then the cells were washed three times with PBS buffer and collected in a centrifuge tube, washed with PBS, and measured the fluorescence intensity with a flow cytometer.

### Determination of intracellular pH

To measure intracellular pH, cells were loaded for 30–60 min with the BCECF-AM with the ratio of 1:1000 and kept in 0.1% hypoxia and normoxia in a 37 °C incubator. The cells were imaged by a fluorescence microscope. Intracellular pH is also measured by flow cytometry using FITC absorbance.

### Molecular docking

Molecular docking studies were carried out using AutoDock Tools 1.5.6 (ADT) and AutoDock Vina 1.1.2. The crystal structure of HIF-1α (PDB ID: 4H6J) and APEX1 (PDB ID: 3U8U) was obtained from the Protein Data Bank. Then, ADT was used to remove all water molecules and co-crystallized ligands from the crystal structure. Subsequently, polar hydrogens and Gasteiger charges were added and the resulted receptors were saved in PDBQT format. The 3D structure of CPM was built, energy minimized, and recorded in Protein Data Bank (PDB) format using Chemoffice software. Then, ADT was used to add polar hydrogens and Gasteiger charges into the CPM and the resulted structure was saved in PDBQT format.

### Wound-healing assay

Cells were seeded in a 12-well plate and allowed to grow to 80% confluency in complete medium. Cell monolayers were scratched by a 200-μL pipette tip, plates were then washed two times with PBS and incubated in medium with targeting compounds for 48 h. Cell migration into the scratched surface was photographed at 0 and 48 h under an inverted fluorescence microscope (×200 magnification).

### Transwell migration assay

Cell were cultured in matrigel-coated membrane with size 8 µm (BD Biosciences, San Diego, CA, USA) with complete medium, treatment was given after 24 h, and kept in 1% O_2_ for 48 h, 30% FBS medium and serum-free medium inside were put then and kept for the next 48 h in 1% O_2_, cells were fixed with 4% paraformaldehyde and stained with 0.5% crystal violet. Filter membrane was examined under a microscope and images were taken.

### Animals and xenograft models

All the experiments were in accordance with the guidelines of the Institutional Animal Care and Use Committee (approval number: 2021-1318). For the experiment male BALB/C nude mice (4–6 weeks of age, weighing 16–20 g) were housed in a specific pathogen-free laboratory animal room. All experiments were performed in accordance with the Animals Care Committee Guidelines. For the initiation of the xenograft model, 200 μL of cell suspension (2 × 10^7^ cells/mL) were implanted subcutaneously into the flank regions of nude mice. After reaching the tumor volume approximately 100 mm^3^, the mice were randomly divided into two groups of 5 each (*n* = 5) and started treating with CPM injection intraperitoneally with 0.4 mg/kg of dose, and the other control group with normal saline. The treatment was continued for 10 days, with a 1-day break after 5 days. The body weights and tumor volumes were recorded every day. The tumor volume (*V*) was determined using the equation: *V* = *A*/2 × *B*^2^, where *A* is the length of the longest side of the tumor and *BM* is the length of the shortest one. Without influencing body organ, each tumor was stored at −80 °C in 0.1% diethylpyrocarbonate solution for western blotting analysis and also fixed in paraformaldehyde for immunohistochemistry.

### Immunohistochemistry assay

Tumor tissues of nude mice are embedded in paraffin, and the sample is cut into 5-μm-thick slices, the sample is treated with citrate buffer and incubated in 3% H_2_O_2_ for 15 min at room temperature, and washed thrice with PBS. The section was blocked with goat serum and incubated with a primary antibody with Ki67 (1:2000), APEX (1:100), HIF-1α (1:200), TXN (1:1000), MMP2 (1:200), and MMP9 (1:200) at 4 °C for 10–12 h. The section is again washed and incubated for 30 min in secondary antibody. It was incubated with diaminobenzidine to produce color section and finally restained with hematoxylin. Images are taken by a fluorescence microscope.

### Statistical analysis

All the statistical analyses were done using ±SEM (Prism 6.0, Graph Pad, La Jolla, CA, USA) and also using one-way ANOVA. To compare the group with the control group Student’s *t*-test was used and significant was set at *p* < 0.05.

## Discussion

As under hypoxia, HIF-1α becomes stable and APEX1 can facilitate the transcription of HIF-1α^29^, and inhibition of APEX1 and its facilitation toward HIF-1α may hamper the APEX1/HIF-1α pathway. The mechanism between APEX1 and HIF-1α brings an opportunity for therapeutic influence^12^. Analysis of publicly available data from TCGA reveals a significant increase in expression of APEX1, HIF-1α, and other targeted proteins in patients. The results of our study support that targeting of APEX1/HIF-1α and disturbing its interaction may lead to the inhibition of LC tumor pH, migration, and angiogenesis. E3330 and LW6 inhibitor can inhibit their specific target, while CPM can inhibit APEX1/HIF-1α pathway as a single agent. The results in this study bring new insights into hypoxia-induced APEX1/HIF-1α pathways in LC.

It was determined that APEX1 in cancer can increase the ROS level due to an unknown mechanism. High expression of ROS level can cause redox imbalance and oxidative stress in tumor cells^30^. The mechanisms underlying ROS regulation in tumor cells are still unclear. Hypoxia brings elevation in ROS levels and abnormalities inside tumor cells^31^. Treatment with E3330 and LW6 can inhibit hypoxia-induced ROS levels in H460 and A549 cells, which confirmed APEX1/HIF-1α mediate hypoxia-induced ROS production. The results from CPM-treated hypoxic cells demonstrate that CPM can inhibit the ROS level under hypoxic condition, probably owing to the inhibition of APEX1/HIF-1α or their interaction.

APEX1 redox activity and CA9 stimulation are important for tumor microenvironmental changes^10^. During hypoxia intracellular pH is one of the key microenvironmental factors that respond to HIF-1α stabilization. An increased intracellular pH promotes the metastatic propagation of tumor cells^24^. The transmembrane protein CA9 responded to hypoxia that regulates intracellular pH by acidifying tumor microenvironment that endorses tumor growth, survival, and invasiveness^7^. CA9 protein expression is inhibited in H460 and A549 cells when treated with CPM at various concentrations in hypoxia. The intracellular pH levels were reduced in hypoxic H460 cells treated with CPM, E3330, and LW6. Collectively, HIF-1α/CA9 regulates intracellular pH and CPM inhibits APEX1/HIF-1α to regulate intracellular pH.

Hypoxic increases the invasions and metastasis potential of tumor cells^8^. Under hypoxic exposure CPM can inhibit the expression of CXCR4 and MMP2 and MMP9 in both A549 and H460 cells, and transwell assay shows that CPM can inhibit the migration of A549 and H460 cells during hypoxia. These results indicate that CPM can inhibit the migration of hypoxic LC cells. Angiogenesis factors were stimulated during hypoxia, which is the main cause of metastasis^32^. We used BAY-86-3934, an EPO inducer, to confirm the response of HUVECs during normoxia. During hypoxia, CPM, E3330, and LW6 significantly inhibit tube formation.

In this paper, we demonstrate that CPM is capable of inhibiting the APEX1 and HIF-1α levels in hypoxic LC cells. Our results revealed that CPM prevents APEX1/HIF-1α colocalization and interaction, thereby leading to the inhibition of elevation of ROS, CA9-mediated intracellular pH, cell migration, and angiogenesis. HIF-1α and APEX inhibition by CPM treatment may improve the treatment efficiency in hypoxic-induced LC cells. Further studies on the novel applications of CPM would help in developing cancer therapies.

## Supplementary information

Supplementary figures
