# Supplementary material for: Cephalomannine inhibits hypoxia-induced cellular function via the suppression of APEX1/HIF-1α interaction in lung cancer
Source: Cell Death Dis. 2021 May 14;12(5):490. doi: 10.1038/s41419-021-03771-z (PMC8121842; doi:10.1038/s41419-021-03771-z)
Supplement: Supplementary file 1 — Supplementary figures [file 41419_2021_3771_MOESM1_ESM.docx]

**Cephalomannine inhibits hypoxia-induced cellular function via the suppression of APEX1/HIF-1α interaction in lung cancer**

Asmat Ullah^1^, Sze Wei Leong ^2^, Jingjing Wang^1^, Qing Wu^1^, Mohsin Ahmad Ghauri^1^, Ammar Sarwar^1^, Qi Su^1,^*, Yanmin Zhang^1,^*

*^1^School of Pharmacy, Health Science Center, Xi'an Jiaotong University, Xi’an, Shaanxi, 710061, P.R. China*

*^2^Department of Microbiology, Faculty of Biotechnology and Biomolecular Sciences, Universiti Putra Malaysia*

Authors Name Email IDs

Asmat Ullah Asmatullah@stu.xjtu.edu.cn

Sze Wei Leong leongszewei@upm.edu.my

Jingjing Wang wjj1714712855@stu.xjtu.edu.cn

Qing Wu wq.xjtu311@stu.xjtu.edu.cn

Mohsin Ahmad Ghauri Ghaurimohsin@stu.xjtu.edu.cn

Ammar Sarwar ammar.pharmacist@stu.xjtu.edu.cn

Qi Su suqi201803@xjtu.edu.cn

Yanmin Zhang zhang2008@xjtu.edu.cn

*Correspondence to:

Dr. Yanmin Zhang, E-mail: zhang2008@mail.xjtu.edu.cn (YM. Zhang)

Dr. Qi Su, E-mail: suqi201803@mail.xjtu.edu.cn

Address: School of Pharmacy, Health Science Center, Xi’an Jiaotong University, No. 76, Yanta West Street, #54, Xi’an, Shaanxi Province 710061, P.R. China

**Figure S1**


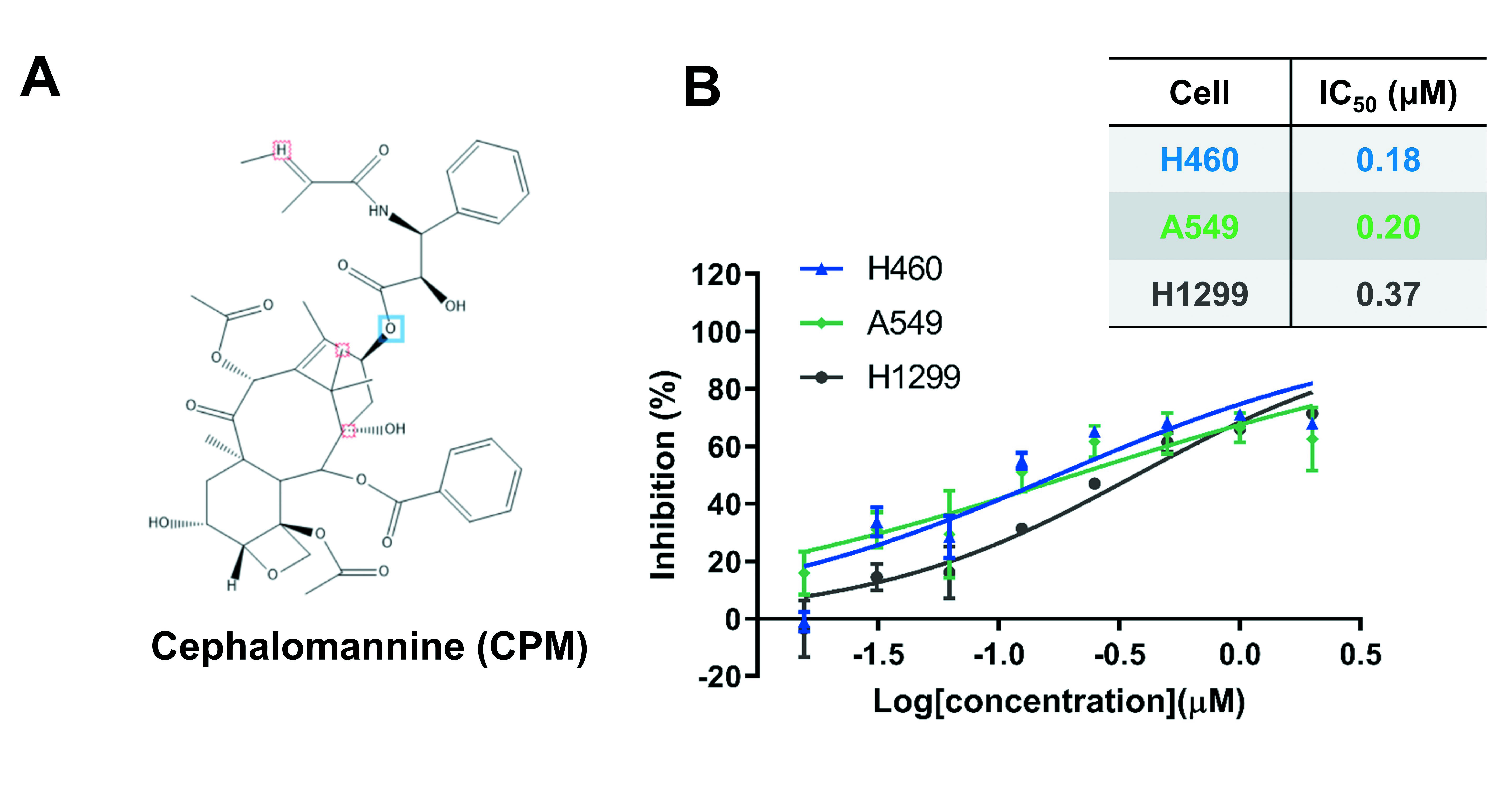


**Supplementary Fig. S1. A** The chemical structure of CPM. **B** Effects of CPM on cell proliferation of LC cells. H460, A549 and H1299 cells were treated with CPM for 48h and cell viability was determined by MTT assay and IC50 values were calculated.

**Figure S2**


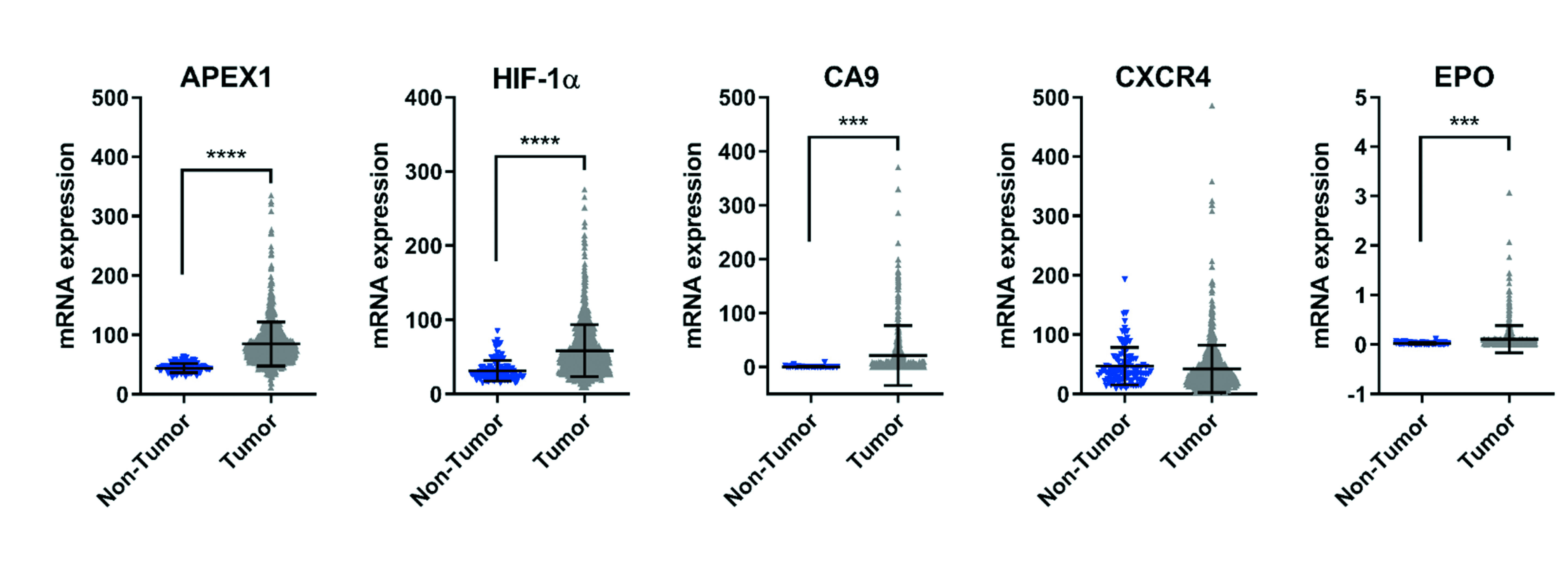


**Supplementary Fig. S2.** APEX1, HIF-1α, CA9, CXCR4 and EPO mRNA expression in Lung adenocarcinoma (533 tumor and 59 non-tumor samples) and squamous cell lung carcinoma (502 tumor and 49 non-tumor samples) analyzed from the TCGA database. ****P* < 0.001, *****P* < 0.0001.
